# Quantitative Analysis of Isoform Switching in Cancer

**DOI:** 10.3390/ijms241210065

**Published:** 2023-06-13

**Authors:** Georgii Dolgalev, Ekaterina Poverennaya

**Affiliations:** Institute of Biomedical Chemistry, Moscow 119281, Russia; dynev.aw@gmail.com

**Keywords:** isoform switching, cancer, differential transcript usage, differential transcript expression, alternative splicing, SatuRn, transcriptomics

## Abstract

Over the past 8 years, multiple studies examined the phenomenon of isoform switching in human cancers and discovered that isoform switching is widespread, with hundreds to thousands of such events per cancer type. Although all of these studies used slightly different definitions of isoform switching, which in part led to a rather poor overlap of their results, they all leveraged transcript usage, a proportion of the transcript’s expression in the total expression level of the parent gene, to detect isoform switching. However, how changes in transcript usage correlate with changes in transcript expression is not sufficiently explored. In this article, we adopt the most common definition of isoform switching and use a state-of-the-art tool for the analysis of differential transcript usage, SatuRn, to detect isoform switching events in 12 cancer types. We analyze the detected events in terms of changes in transcript usage and the relationship between transcript usage and transcript expression on a global scale. The results of our analysis suggest that the relationship between changes in transcript usage and changes in transcript expression is far from straightforward, and that such quantitative information can be effectively used for prioritizing isoform switching events for downstream analyses.

## 1. Introduction

Dysregulation of alternative splicing and other RNA-level processes is one of the signature features of cancer [1,2,3]. Ultimately, this dysregulation results in altered levels of individual transcripts, some of which may exert disease-relevant functions. It is now well known that transcripts originating from the same gene can serve different and sometimes even antagonistic biological roles [4,5]. A prominent example of the latter is a human gene *BCL2L1*, which encodes two proteins, BCL-X_L_ and BCL-X_S_, that differ due to the presence of alternative 5′ splice sites in the second exon of the gene [6]. While BCL-X_L_ has an antiapoptotic function and is frequently upregulated in cancer [7], the shorter isoform BCL-X_S_ promotes cell death [8,9]. Accordingly, transcript-level information can yield important insights into cancer biology that might have otherwise been missed in gene-level analyses.

One particularly interesting approach to leveraging this information is concerned with detecting so-called isoform switching events, in which one transcript is upregulated and another transcript from the same gene is downregulated in tumor tissue in comparison to normal tissue. The first study of this phenomenon, which essentially introduced the concept of isoform switching, was published in 2015 by Sebestyén et al. [10]. The definition of isoform switching in that study was primarily based on the frequency of the switch, requiring two transcript isoforms to reverse the order of prevalence in a sufficient proportion of tumor samples to be considered as an isoform switch. Additionally, the last stage of their analysis required that the two transcripts involved in a switch had an inverse correlation between their transcript usage values, which is a proportion of the transcript’s expression in the overall expression of the parent gene (also known as isoform fraction, IF [11], or percent spliced in, PSI [10,12]). Two years later, a very different approach to detecting isoform switching was presented by Climente-Gonzalez et al., who focused on individual tumor samples where a change in a transcript’s usage in cancer was larger than the variation of the transcript’s usage in normal samples [12]. This strategy led to the detection of a very high number of mostly lowly recurrent switches. Another study, published in the same year by Vitting-Seerup et al., offered a simple but powerful strategy for detecting recurrent isoform switches [11]. Briefly, isoform switching was defined as an event in which one transcript isoform had a mean change in transcript usage between two conditions higher than 0.1, and this change was statistically significant (as measured by Mann–Whitney U-test), while another transcript from the same gene had a statistically significant mean change in transcript usage of −0.1 and less. As multiple transcripts from the same gene could be upregulated and downregulated in this manner at the same time, each gene could theoretically have multiple switches per cancer type, and one transcript could be involved in multiple switches as well. With this approach, the authors identified 4446 switches with predicted functional consequences in 12 cancer types. In addition to identifying switches, this study presented an R library, IsoformSwitchAnalyzeR, which was used to identify global changes in features between upregulated and downregulated transcripts.

Although several alternative approaches to analyzing isoform switching in cancer have been presented [13,14], the general strategy proposed by Vitting-Seerup et al. and implemented as part of IsoformSwitchAnalyzeR remains the most popular approach to identifying isoform switching events. For example, it has been used to detect isoform switching in sepsis [15], in esophageal adenocarcinoma [16], and in Alzheimer’s disease-affected human brains [17]. Since the initial study, the part of IsoformSwitchAnalyzeR responsible for assessing differential transcript usage has been upgraded several times with more accurate and performant DTU tests implemented in DEXSeq [18] or DRIMSeq [19], but the general strategy remained the same.

It is interesting that most of the studies of isoform switching relied, at least in part, on transcript usage, rather than transcript expression, to identify isoform switching events. The relationship between changes in transcript usage and changes in transcript expression is not straightforward; for example, a differentially expressed transcript might not be differentially used and vice versa [20]. Since expression is a much more biologically relevant quantity than transcript usage, we reasoned that examining isoform switching from the quantitative point of view can provide additional information to rank switching events and decrease the number of potentially interesting switches from several thousand to a much more manageable number.

To this end, we adopted the definition of isoform switching as proposed by Vitting-Seerup et al. and used a novel algorithm for the analysis of differential transcript usage, SatuRn [21], to identify isoform switches in 12 cancer types on the basis of RNA-Seq data from TCGA project. After identifying the switches, we analyzed how detected changes in transcript usage correlate with changes in expression for the affected transcripts and how often these pairs of transcripts changed the order of prevalence between cancer and normal tissue. Our results surprisingly revealed that only in a third of the identified switches both transcripts had the same direction of change in transcript usage as in transcript expression, and that switches where one transcript almost always had a higher expression than the other transcript were common. These observations detail the landscape and mechanisms of transcriptional dysregulation in cancer by uncovering a new dimension of the phenomenon of isoform switching, which could additionally be used to improve functional inferences from differential transcript usage-based isoform switching analyses.

## 2. Results

### 2.1. Detection of Isoform Switching in Cancer Using SatuRn

Similar to other investigations of isoform switching in cancer, we relied on RNA-Seq data from TCGA project, which, in our case, was acquired from the recent reanalysis performed as part of the UCSC Toil Recompute project [22]. For our study, we selected 12 cancer types that had data for at least 25 normal samples (Table S1).

We adopted the general definition of isoform switching as proposed by Vitting-Seerup et al. [11] and first analyzed differential transcript usage in each cancer type with the help of SatuRn. Unique features of SatuRn include good control of false discovery rate and speed [21], which was necessary as most of the selected cancer types had several hundreds of samples. After testing for differential transcript usage and extracting transcripts with significant changes in usage, we analyzed whether these transcripts were sufficiently upregulated or downregulated by comparing their mean usage values between cancer and normal samples. If the mean change in transcript’s usage value was higher than 0.1, the transcript was considered upregulated; if less than −0.1, it was considered downregulated. The results of testing for differential transcript usage and other quantitative data for individual transcripts can be found in Table S2. Lastly, for a given gene in a given cancer type, isoform switching events were identified by pairing statistically significant upregulated transcripts with statistically significant downregulated transcripts in all possible combinations. To prevent ambiguity, since regulation can be formulated in terms of both usage and expression, from here on, we call transcripts upregulated in terms of usage as “cancer” transcripts and transcripts downregulated in terms of usage as “normal” transcripts.

**Figure 1:**
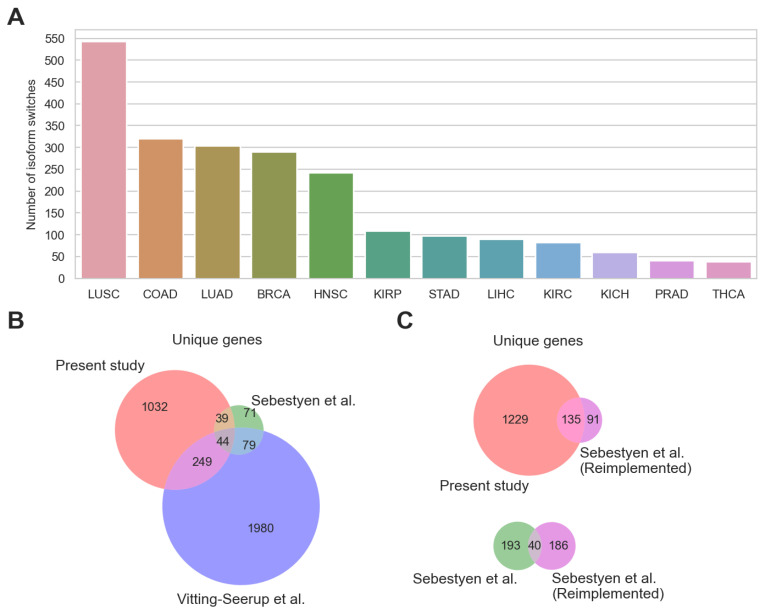
General results. (**A**) The number of identified isoform switches per cancer type. (**B**) Overlap of unique genes identified in the present study, Sebestyén et al. study [10], and Vitting-Seerup et al. study [11]. (**C**) Overlap of unique genes identified in the present study and Sebestyén et al. [10] approach applied to the same dataset.

In total, we identified 2217 isoform switches (Table S3), with numbers varying considerably across different cancer types (Figure 1A). Identified switches affected 1364 unique genes and included 1605 unique pairs of transcripts.

To find out how our results compared to the previous investigations of recurrent isoform switching in cancer, we analyzed the overlap of our results, the Sebestyén et al. study, and the Vitting-Seerup study in terms of unique genes affected by isoform switching (note that Vitting-Seerup et al. considered the same 12 cancer types as in this study, while Sebestyén et al. considered three fewer cancer types) (Figure 1B). The overlap of our study and the studies of Sebestyén et al. and Vitting-Seerup et al. was low; only 44 genes were shared across all three (Figure 1B). What was more surprising was that the overlap of the present study and the Vitting-Seerup et al. study was small despite overall similar strategies; only 293 genes were shared between the studies (less than 23% of unique genes identified in the present study). Due to the fact that not only strategies for isoform switching detection differed between the studies, but also details of RNA-Seq analysis and annotation databases used, it was hard to pinpoint the exact reason for the discrepancy. In an attempt to at least partially resolve this question, we managed to fully reconstruct the analysis pipeline used by Sebestyén et al. (see Section 4) and applied it to our dataset. The share of unique genes identified using the reconstructed algorithm and also identified in our analysis increased from 38% to 60% in comparison with the initial study (Figure 1C). Remarkably, overlap between the original results of Sebetyen et al. and the results obtained after applying their pipeline to our dataset was also quite low; only 40 unique genes were shared between them (approximately 17% of each result in terms of unique genes). Therefore, we cautiously argue that the choice of annotation and RNA-Seq analysis strategy might in fact account for a significant portion of discrepancy between studies investigating isoform switching, something that has been suggested before [23]. However, due to the large differences between studies in terms of methodology and technical aspects, proving this statement for certain is a tough task outside of the scope of the present work.

### 2.2. Relationship between Changes in Transcript Usage and Changes in Transcript Expression

**Figure 2:**
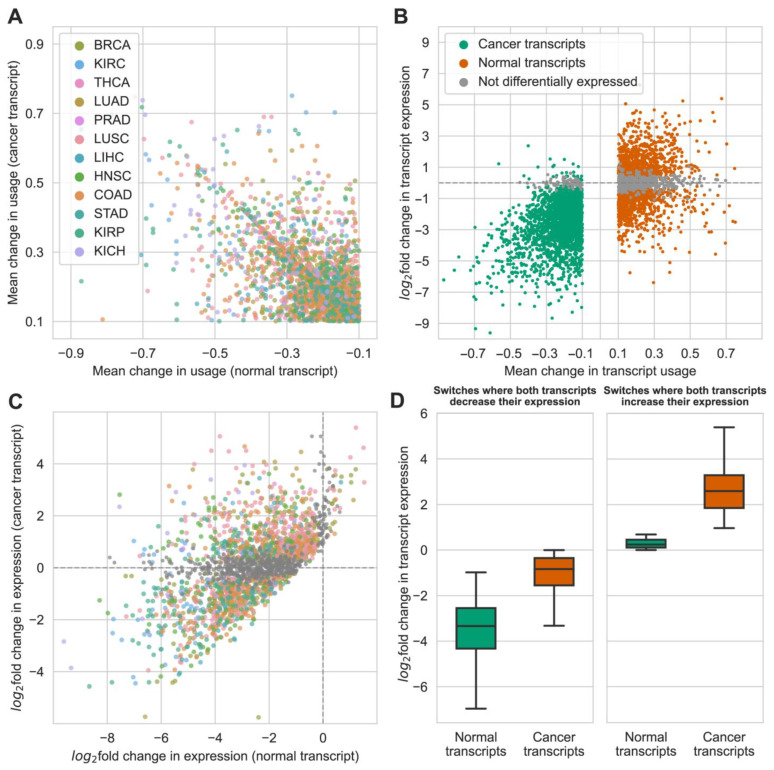
Relationship between change in transcript usage and change in transcript expression for the identified isoform switches. (**A**) Scatterplot of mean changes in usage of transcripts participating in isoform switching. (**B**) Relationship between mean change in transcript usage and fold change of expression for cancer (upregulated in terms of usage) and normal (downregulated in terms of usage) transcripts separately. Transcripts that are not differentially expressed are colored gray. (**C**) Scatterplot of fold changes of expression of transcripts participating in isoform switching. The color scheme is inherited from (**A**,**B**). (**D**) Comparison of fold changes of expression between normal and cancer transcripts from isoform switches in which both transcripts either decreased or increased their expression levels.

Before we could move to analyze the interplay between transcript usage and transcript expression for the identified isoform switches, an understanding of the distribution of changes in isoform usage was required to set a background for our analysis. A scatterplot of mean changes in transcript usage did not point to any particular anomalies (Figure 2A). However, it was clear that most of the identified isoform switches were composed of transcripts with only a modest change in usage; approximately 50% of switches had a mean change in usage from −0.1 to −0.24 for the normal transcript and a mean change in usage from 0.1 to 0.24 for the cancer transcript. Additionally, while a statistically significant difference was observed between mean changes in transcript usage between cancer types (cancer transcripts: adj. *p* < 1.47 × 10^−21^; normal transcripts: adj. *p* < 3.24 × 10^−38^, Kruskal–Wallis H-test), these differences were not extreme in magnitude (Figure S1). Furthermore, only six cancer types (KIRC, LUAD, LUSC, LIHC, COAD, and KIRP) out of 12 had statistically significant differences in absolute mean changes in transcript usage between cancer and normal transcripts (adj. *p* < 0.05, Wilcoxon signed-rank test). These observations justified further inference from all identified switches as a single population.

Having established the overall pattern of changes in transcript usage, we moved on to explore how changes in transcript usage were connected to changes in transcript expression for the affected transcripts. To this end, we calculated log_2_-transformed fold changes in expression and performed differential expression testing for all transcripts which were initially considered for differential transcript usage (Table S2). Note that we did not take fold change into account when considering whether a transcript is differentially expressed or not, and only considered the results of the statistical testing. A plot of mean changes in transcript usage versus log_2_-transformed fold changes of expression levels of individual transcripts revealed interesting results (Figure 2B). First, a significant number of transcripts, 515 (24.6%) cancer transcripts and 76 (3.6%) normal transcripts that were differentially used, were in fact not differentially expressed. This means that changes in the usage of these transcripts were caused by changes in the expression of other transcripts from the same gene, most likely the second transcript in the switch. Interestingly, while normal transcripts that were not differentially expressed had a lower mean change in usage than differentially expressed normal transcripts (adj. *p* < 0.0019, one-sided Mann–Whitney U-test), there was no difference in terms of mean changes in usage between differentially expressed and not differentially expressed cancer transcripts (adj. *p* = 0.52, two-sided Mann–Whitney U-test). We did not observe any isoform switches in which both transcripts were not differentially expressed, which is consistent with the definition of transcript usage (if expression levels of two transcripts from the same gene stay the same across conditions, their change in usage will be equal and cannot be opposite). Secondly, a large number of transcripts that were differentially expressed had an opposite direction of change in expression relative to change in usage. This phenomenon was much more pronounced in the case of cancer transcripts; more than half (53.4%) of differentially expressed cancer transcripts were upregulated in terms of usage, but downregulated in terms of expression, while the inverse was true for only 1.4% of normal transcripts. Note that, since we analyzed normal and cancer transcripts separately, each transcript was counted only once per cancer type for calculating the percentages. As with transcript usage, there were cancer type-specific differences in terms of the average change in expression levels (cancer transcripts: adj. *p* < 6.51 × 10^−17^, normal transcripts: adj. *p* < 6.84 × 10^−78^, Kruskal–Wallis H-test), but the overall pattern was shared across all types; each cancer type had switches where one of the transcripts was not differentially expressed and switches where one of the transcripts had changes in expression opposite to changes in usage (Figure S2). Thus, the distributions of both cancer-specific changes in transcript usage and changes in transcript expression for switch-affected transcripts support pooling all the identified switches for our analysis.

Summarizing these results, out of 2217 initially identified switches, only 1602 of them (72.3%) had both transcripts that were differentially expressed (Figure 2C). The remaining switches were composed of pairs of transcripts in which one of them had no statistically significant change in expression between normal and cancer samples. Out of 1602 switches where both transcripts were differentially expressed, only 802 (36.2% of the total number of switches) had changes in expression consistent with the direction of changes in usage. The majority of the remaining switches, 771 (34.8% of the total number of switches) had both transcripts decreasing their expression in cancer samples, and the 29 (1.3%) remaining switches had both transcripts increasing their expression. Although this seemed counterintuitive, a closer inspection provided an explanation; in the case where both transcripts in a switch decreased their expression in cancer samples, normal transcripts on average had a more significant decrease in expression (adj. *p* < 8.58 × 10^−127^, one-sided Wilcoxon signed-rank test), which compensated for the decrease in expression of cancer transcripts (Figure 2D). The same logic was true for switches in which both transcripts increased their expression; in these cases, normal transcripts on average had a significantly smaller increase in expression than cancer transcripts (adj. *p* < 1.28 × 10^−6^, one-sided Wilcoxon signed-rank test). No isoform switches had a cancer transcript that decreased its expression level and a normal transcript that increased its expression level in cancer cells.

### 2.3. Influence of the Level of Transcript Expression on the Magnitude of Changes in Usage or Expression

**Figure 3:**
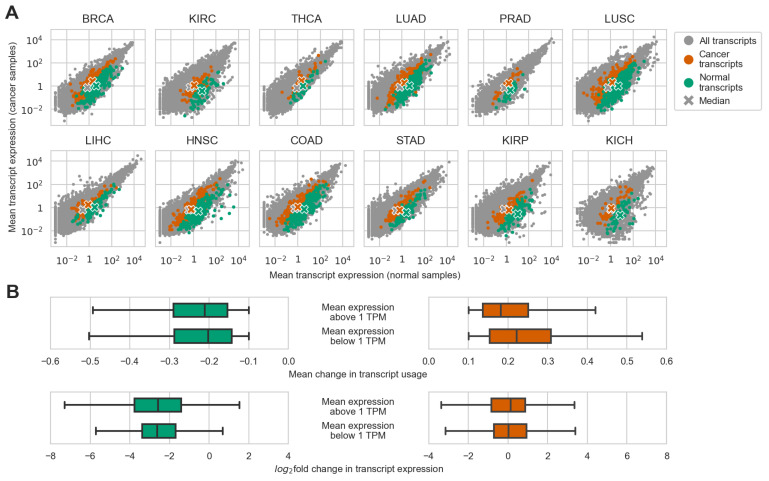
Relationship of changes in transcript usage, transcript expression, and baseline expression level of transcripts affected by isoform switching. (**A**) Expression of transcripts participating in isoform switching in contrast to all transcripts considered per cancer type. (**B**) Comparison of changes in usage and fold changes of expression between lowly and highly expressed transcripts for all the identified isoform switches.

Considering these rather unintuitive results, we wondered if some of them could have been caused by transcripts or genes with a low baseline expression level, for which even a small change in expression would cause a significant change in transcript usage or a large fold change in expression. To answer this question, we first simply analyzed the distribution of mean expression of normal and cancer transcripts versus all transcripts considered for differential usage per cancer type. A brief look at the resulting plots revealed that both normal and cancer transcripts did not belong to transcripts with extremely low expression levels in both normal and cancer samples (Figure 3A). To assess these relationships statistically, we compared the expression levels of both normal and cancer transcripts against the expression levels of all transcripts considered for DTU in both normal and cancer samples. For almost all cancer types, both normal and cancer transcripts had an expression level in normal samples that was greater than all transcripts per cancer type (adj. *p* < 0.05, one-sided Mann–Whitney U-test) with the exception of cancer transcripts in KICH (adj. *p* = 0.32). The results become less predictable when considering the expression of switch-affected transcripts in cancer samples. Six cancer types (BRCA, THCA, LUAD, LUSC, LIHC, and COAD) out of 12 had switch-affected cancer transcripts with a generally higher expression level than all transcripts in the respective cancer samples (adj. *p* < 0.05, one-sided Mann–Whitney U-test), while only three cancer types (LUAD, LUSC, and LIHC) had switch-affected normal transcripts with a higher expression level than all transcripts in the respective cancer samples (adj. *p* < 0.05, one-sided Mann–Whitney U-test).

These results were generally consistent with our previous observations; half of the cancer transcripts demonstrated a decrease, rather than an increase, in expression, which led to them being expressed at least not higher than all transcripts in cancer samples in several cancer types.

Nevertheless, in all cancer types, there were some switch-affected transcripts that had a lower expression level than the median expression level for all transcripts. As such, it might be possible that these transcripts contributed to the anomalous results. To test this hypothesis, we first calculated the Spearman correlation between transcript expression levels in normal samples (as all changes happen relative to them) and changes in transcript usage or fold changes in expression for normal and cancer transcripts separately. No apparent strong correlation was observed in all cases (absolute Spearman’s ρ < 0.29). We then separated all normal and cancer transcripts into two groups each on the basis of whether their expression in normal samples was lower or higher than a threshold. We set the threshold to 1 TPM, which is a common value for separating lowly and highly expressed transcripts [10]. Next, we tested whether there were differences between levels of change in mean transcript usage and log_2_-transformed fold changes in expression (Figure 3B). A statistically significant difference was detected only between changes in mean transcript usage between lowly and highly expressed cancer transcripts (adj. *p* < 0.05, Mann–Whitney U-test), but the difference was small (difference between medians of highly and lowly expressed transcripts = −0.04). Therefore, there was no significant evidence that lowly expressed transcripts could have skewed the results.

### 2.4. Frequency of the Detected Switches

In contrast to the algorithm used by Sebestyén et al. [10], two transcripts do not necessarily have to switch the order of prevalence between normal and cancer samples for them to be considered switching in the present analysis. However, it was still interesting to see how the identified switches are ranked in terms of this parameter, as such behavior is more intuitively associated with the concept of “switching”, and it can be argued that pairs of transcripts with a high rate of prevalence switching are more interesting for further analysis.

**Figure 4:**
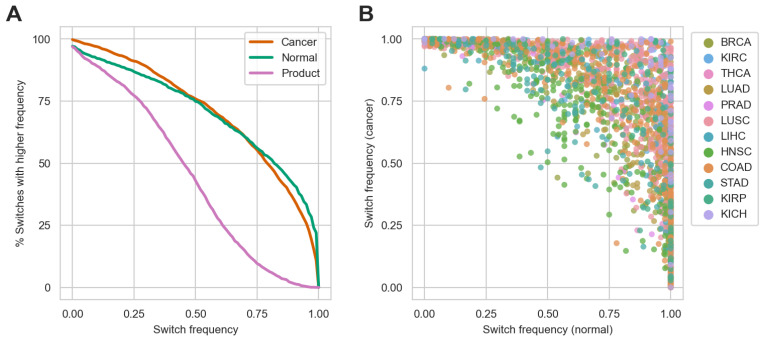
Frequency of change in the order of prevalence for the detected isoform switches. (**A**) The proportion of identified switches for which one of the characteristic frequencies was higher than a given frequency. “Product” stands for a combined frequency, which for a given switch is a product of its two characteristic frequencies. (**B**) Scatterplot of characteristic frequencies for all isoform switching events.

Each isoform switching event could be characterized by two sub-frequencies—the “cancer” frequency, which measures the proportion of cancer samples in which the cancer transcript has a higher expression than the normal transcript, and the “normal” frequency, which measures the proportion of normal samples where the normal transcript has a higher expression than the cancer transcript (Table S3). Note that the definition of these frequencies is not influenced by the use of either transcript expression or transcript usage for calculations, as the resulting frequencies would be the same. We were interested in the proportion of identified isoform switches that had each of these sub-frequencies higher than a certain percentage, e.g., 50% or 75% (which is equal to transcripts appropriately switching the order of prevalence in 50% or 75% of samples, respectively) (Figure 4A). The resulting curves indicated that transcripts from roughly 75% of isoform switches appropriately switched the order of prevalence in at least 50% of either cancer or normal samples. However, the proportion of switches with one of the frequencies higher than a given value started to fall more quickly with the increase in the given frequency.

Since each isoform switching event had two characteristic frequencies, the interplay between them was found to be complicated (Figure 4B). While 51% of switches had both sub-frequencies higher than 0.5, and as 16% of switches had both sub-frequencies higher than 0.75, we identified 50 cases in which isoform switches had one of the frequencies equal to 1.0 and the other equal to 0, meaning that one of the involved transcripts had a higher expression than the other in all samples in both normal and cancer samples.

Accordingly, a combined score based on these two frequencies is needed to unambiguously rank the identified isoform switches. Sebestyén et al. used a sum of the characteristic frequencies minus one. However, such a sum would be equal for a switch with frequencies (1.0, 0.0) and a switch with frequencies (0.5, 0.5). Thus, we reasoned that a score that favors switches like the latter rather than the former would be more appropriate. As such, we calculated a combined frequency for each switch as a product of its two sub-frequencies. The resulting curve indicates that approximately 40% of switches had a combined frequency of 0.5 and higher, which roughly corresponds to isoform switches with both sub-frequencies equal to 0.7 or similar and higher (Figure 4A).

### 2.5. Ranking Isoform Switching Events Using Expression and Frequency Data

Our quantitative analysis gave us the information required to further prioritize the identified isoform switches. While the exact value of assayed parameters for prioritizing switches is a subject for discussion, and while individual cases must be treated in an individual manner, for our global analysis, we reasoned that a switch is more likely to include transcript isoforms with opposite behavior and, thus, probably functionality, if it abides by several rules. First, both transcripts in a switch must be differentially expressed; second, the direction of the change in the expression must be codirectional with the change in usage for each transcript. The resulting list of 802 switches (36.2% of the initial result) can be further filtered by a combined frequency of the switch. For instance, we might want to remove switches with a combined frequency of 0, representing switches where one transcript always has a higher expression than the other transcript. After removing such cases, we were left with 776 switches (35.0% of the initial list of switches). Finally, switches in the resulting list can be ranked by combined frequency. Additional filtering, for example, by requiring the combined frequency to be higher than 0.5 would further shorten the list, leaving only 375 switches (16.9%). Alternatively, the switches could be filtered in terms of log_2_ fold changes of expression of the affected transcripts by, for instance, requiring that absolute log_2_ fold changes of expression must be higher than 1 (meaning twofold increase or decrease) for each transcript, as commonly required by differential expression workflows. However, even without applying such filters, the reduction in switch numbers was already quite pronounced. Therefore, even the most basic filtering criteria could effectively reduce the number of interesting switches threefold, significantly decreasing the burden of further functional analysis of the switching consequences.

## 3. Discussion

The present work serves as both an original inquiry into the phenomenon of isoform switching in cancer and a reflection of previous work in this area of research. Unfortunately, there is currently no universally accepted definition of isoform switching as different studies introduce different methodologies tailored to answering specific biological questions. In the present study, we focused on a more commonly used definition of isoform switching that is based on differential transcript usage; however, as we already mentioned, multiple studies also used transcript usage in a different capacity to identify switches.

Although there are plenty of tools for detecting differential transcript usage, this concept is frequently confusing for researchers new to this topic, primarily because of the complicated relationship of differential transcript usage, differential transcript expression, and differential gene expression [24,25,26]. In the present study, we demonstrated that this confusion is justified, and significant implications arise with respect to the interpretation of the results of differential transcript usage analysis and isoform switching in particular, as it has been one of the most insightful applications of DTU.

Using SatuRn to perform robust and accurate testing for differential transcript usage in 12 human cancer types, we confirmed previous reports of a high number of isoform switches in human cancers. However, our subsequent analysis showed that only one-third of the identified switches were composed of transcripts which both have the same direction of change in their usage compared to the change in expression, and the magnitude of these changes was not correlated. In approximately half of the rest of the identified switches, one of the transcripts did not change its expression across conditions, implying that these events are better described as single-transcript differential expression events, which has implications for functional analysis of their consequences. The final third of isoform switches was composed of transcripts that both decreased (or increased) their expression, despite having opposite changes in usage. As such, these transcripts were both downregulated (or upregulated) in terms of expression, providing evidence for their hypothetical joint, and not alternative, regulation. We additionally made an observation that, while most of the normal transcripts in the identified isoform switches were downregulated in terms of both usage and expression, cancer transcripts on the other hand were predominantly either not differentially expressed or also downregulated (in terms of expression), meaning that isoform switches were closely associated with the overall downregulation in gene expression in cancer.

Do these observations invalidate transcript usage as a measure of alternative regulation of transcripts? Certainly not, as several lines of evidence suggest that a mere change in transcript ratios can trigger important biological processes [27,28,29], and this phenomenon is particularly pronounced in apoptosis, in which many of the associated genes encode protein isoforms with distinct or opposite functions [30,31,32,33]. This means that differential expression of both transcripts is not required for these changes to take place. However, such changes quite often depend on the actual switching of the order of prevalence between the major and the minor transcript [34]. As we also showed in this article, this does not happen consistently for a significant portion of the identified switches; for example, we discovered cases where the normal transcript always had a higher expression than the cancer transcript despite the former being downregulated and the latter upregulated.

Summarizing the results, our analysis indicates that the pronounced transcriptional dysregulation in cancer, previously phrased in terms of opposite changes in proportions of transcripts, i.e., isoform switching, is for the large part caused by the overall downregulation of the switch-affected transcripts in terms of expression. From the methodological standpoint, we recommend always supplementing differential transcript usage-based workflows such as isoform switching analysis with transcript expression data. While the interpretation of all of these data and their relative value depends on the specific pair of transcripts and the biological question addressed, these quantitative data can provide additional information to prioritize isoform switches and conduct an adequate assessment of the functional consequences of the switch.

## 4. Materials and Methods

### 4.1. General

DTU and DTE analyses were performed in R v.3.2.4. General data analysis and plotting were performed in Python v.3.11. Correction for multiple testing was performed with the Benjamini–Hochberg procedure as implemented in the statsmodels Python library. FDR-adjusted *p*-values < 0.05 were considered significant.

### 4.2. Data

TCGA RNA-Seq data were quantified as part of the UCSC Toil RNA-Seq Recompute project [22] using kallisto [35] and GENCODE human release 23 annotation (GRCh38) [36]. Transcript-level counts, TPM values, and sample metadata were downloaded from TCGA Pan-Cancer (PANCAN) dataset page (https://xenabrowser.net/datapages/?cohort=TCGA%20Pan-Cancer%20(PANCAN), accessed on 11 December 2022) using Xena Browser [37]. Only cancer types with at least 25 normal samples were selected for further analysis. The GENCODE human release 23 Comprehensive gene annotation file was downloaded from the GENCODE archive.

### 4.3. Analysis of Differential Transcript Usage

SatuRn v.1.7.3 [21] was used to test for differential transcript usage. Briefly, count-level data were filtered using edgeR’s [38] expression filter with default parameters. Differential transcript usage was calculated using SatuRn’s fitDTU and testDTU functions with the same parameters as in the tool’s manual. The results for each cancer type were aggregated in one table, and global correction for multiple testing was performed on empirical *p*-values produced by SatuRn.

### 4.4. Identification of Isoform Switches

Transcript usage values were calculated from TPM values by dividing the expression level of each transcript by the expression level of its parent gene in each sample. In samples where the gene was not expressed, transcript usage values were set equal to 0. The mean change in transcript usage was calculated for each transcript in each cancer type by subtracting mean transcript usage in normal samples from mean transcript usage in cancer samples. For a particular gene in a particular cancer type, isoform switches were composed of all possible combinations between upregulated (mean transcript usage > 0.1, DTU adj. *p* < 0.05) and downregulated (mean transcript usage < −0.1, DTU adj. *p* < 0.05) transcripts.

### 4.5. Analysis of Differential Transcript Expression

edgeR v.3.40.2 [38] was used for differential expression testing. Briefly, count-level data were filtered using edgeR’s expression filter with default parameters (essentially, the same transcripts were tested for both DTU and DTE). Scaling for library size was performed with edgeR’s default algorithm, TMM. Dispersions were estimated using the estimateCommonDisp function, and differential expression testing was performed using the glmQLFit and glmQLFTest functions with default parameters. The results for each cancer type were aggregated in one table, and global correction for multiple testing was performed.

### 4.6. Reconstruction of Sebestyén et al.’s Approach to Isoform Switching Detection

The iso-kTSP v.1.0.3 tool [10] was downloaded from Bitbucket (https://bitbucket.org/regulatorygenomicsupf/iso-ktsp/downloads/, accessed on 21 December 2022), and Java v.8.361 was used to run the package. Expression data were formatted accordingly to the format used by the authors of the study, and the example datasets were downloaded from figshare (https://bitbucket.org/regulatorygenomicsupf/iso-ktsp/downloads/, files with the suffix “iso_iktsptpm_paired-normal-tumor-filtered”, accessed on 21 December 2022). Identification of isoform switches was performed with the iso-kTSP tool using the following parameters: -i (for transcript-level analysis), -k = 50, and -n set equal to the number of normal samples in the supplied dataset. A permutation test for each cancer type was performed on the same dataset as used for the previous step with the parameters -i and -l = 1000. Significant isoform switches were derived from the ranking step by requiring that score1 and information gain (IG) of the significant switches must be higher than the maximum values of these quantities obtained in the permutation step. The resulting list of significant switches was further filtered by expression levels of the switch-affected transcripts, requiring both transcripts in the switch to have a mean expression of 1 TPM in either cancer or normal samples. Lastly, only switches in which the transcripts had a negative correlation between their transcript usage values (Spearman’s ρ < −0.8) were left. The resulting pipeline was tested on three randomly chosen cancer types (KICH, COAD, and PRAD) using the datasets provided by the authors (see above). The results of applying this reconstructed pipeline to the subset of cancer types produced exactly the same results as reported by Sebestyén et al. (Supplementary File S4 in the original study, file “tcga_isoform_switches”).

## Data Availability

Data generated and used in this study are available in the Supplementary Materials.

## Supplementary Materials

The following supporting information can be downloaded at https://www.mdpi.com/article/10.3390/ijms241210065/s1.
