# Supplementary material for: Quantitative Analysis of Isoform Switching in Cancer
Source: Int J Mol Sci. 2023 Jun 13;24(12):10065. doi: 10.3390/ijms241210065 (PMC10298363; doi:10.3390/ijms241210065)
Supplement: Supplementary file 1 [file ijms-24-10065-s001.zip › ijms-2380963-supplementary/ijms-2380963-supplementary-figures.pdf]

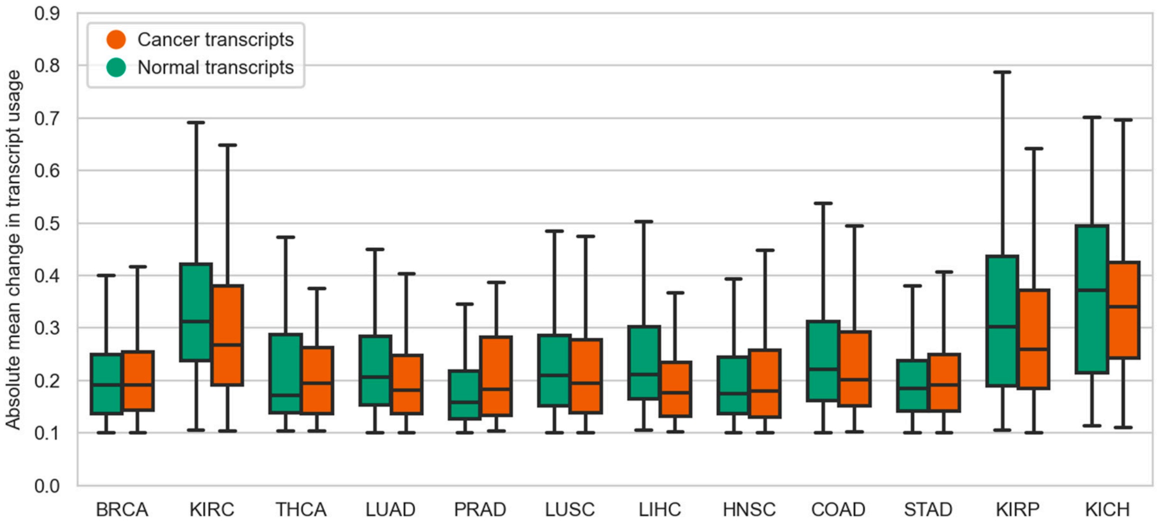

Figure S1. Comparison of mean changes in transcript usage in the identified isoform switches between cancer types.

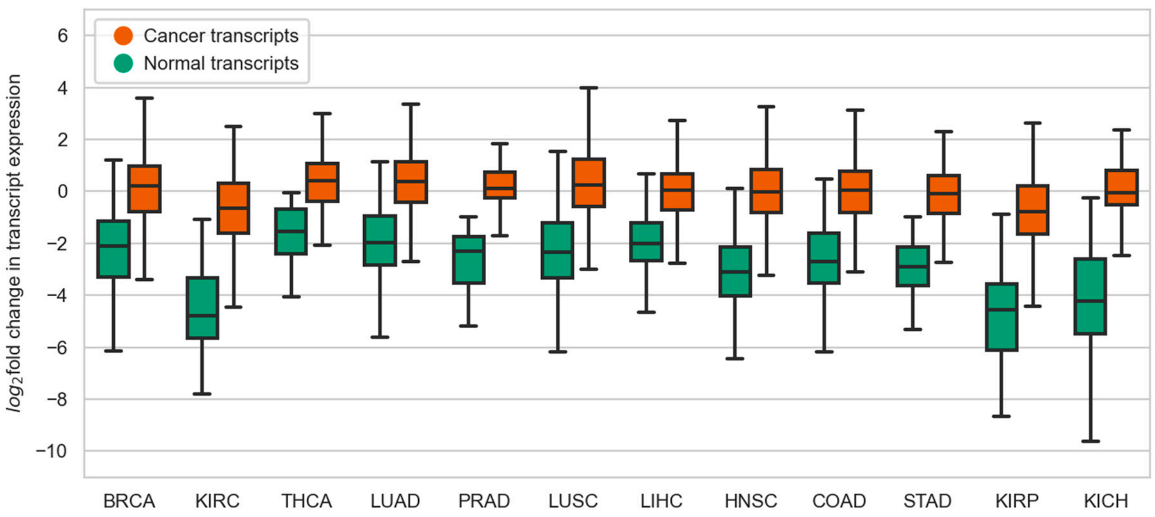

Figure S2. Comparison of log2 fold changes of transcript expression in the identified isoform switches between cancer types.
